# Association of Neuraxial Labor Analgesia for Vaginal Childbirth With Risk of Autism Spectrum Disorder

**DOI:** 10.1001/jamanetworkopen.2021.40458

**Published:** 2021-12-22

**Authors:** Loreen Straub, Krista F. Huybrechts, Helen Mogun, Brian T. Bateman

**Affiliations:** 1 Division of Pharmacoepidemiology and Pharmacoeconomics, Department of Medicine, Brigham and Women’s Hospital, Harvard Medical School, Boston, Massachusetts; 2 Department of Anesthesiology, Perioperative and Pain Medicine, Brigham and Women’s Hospital, Harvard Medical School, Boston, Massachusetts; 3 Department of Anesthesiology, Perioperative and Pain Medicine, Stanford University School of Medicine, Stanford, California

## Abstract

**Importance:**

Recent studies have reported conflicting findings regarding a potential association between analgesia used during labor and autism spectrum disorder in the offspring.

**Objective:**

To evaluate whether neuraxial labor analgesia increases the risk of autism spectrum disorder in the offspring.

**Design, Setting, and Participants:**

This cohort study included mother-child dyads who underwent vaginal delivery and were exposed to neuraxial labor analgesia. Delivery data were collected from the Medicaid Analytic eXtract (2005-2014) for mothers with public insurance and the IBM Health MarketScan Research Database (2005-2015) for mothers with private insurance. Data analysis was conducted from January to October 2021.

**Exposures:**

Presence of a procedure code indicating neuraxial labor analgesia.

**Main Outcomes and Measures:**

Children with autism spectrum disorder, identified using a validated algorithm (positive predictive value: 94% [95% CI, 83%-99%]). Cumulative incidence curves stratified by exposure were assessed using Kaplan-Meier analyses. Hazard ratios were estimated through Cox proportional hazards regression, using propensity-score fine stratification for confounding control. Estimates from both insurance cohorts were combined through fixed-effects meta-analysis. Subsequently, results from these analyses were combined with existing published studies.

**Results:**

The cohort of mother-child dyads with public insurance consisted of 910 696 deliveries (mean [SD] maternal age, 24.3 [5.7] years; 286 025 [31.4%] Black mothers; 374 282 [41.1%] White mothers), with 484 752 (53.2%) being exposed to neuraxial labor analgesia. The cohort of mother-child dyads with private insurance included 696 883 deliveries (mean [SD] maternal age, 31.0 [4.5] years; race and ethnicity data not available), with 513 347 (73.7%) being exposed. Cumulative incidence of autism spectrum disorder by 10 years of age was 1.93% (95% CI, 1.73%-2.13%) among children in the exposed group vs 1.64% (95% CI, 1.51%-1.76%) among children in the unexposed group in the publicly insured cohort. Respective numbers were 1.33% (95% CI, 1.19%-1.46%) and 1.19% (95% CI, 0.99%-1.38%) in the privately insured cohort. Adjusting for potential confounders and pooling across both cohorts resulted in a hazard ratio of 1.08 (95% CI, 1.02-1.15). Results were consistent when additionally adjusting for empirically identified variables through high-dimensional propensity score analyses (pooled hazard ratio, 1.07; 95% CI, 1.00-1.14) or expanding the cohorts to include cesarean deliveries and assisted vaginal deliveries (pooled hazard ratio, 1.07; 95% CI, 1.03-1.12). Meta-analysis of this study and recently published observational studies yielded similar findings with a pooled hazard ratio of 1.10 (95% CI, 1.06-1.13).

**Conclusions and Relevance:**

Although a small increase in risk cannot be ruled out, the combined evidence from observational studies does not support the notion that neuraxial labor analgesia is associated with an increased risk of autism spectrum disorder.

## Key Points

**Question:**

Does neuraxial labor analgesia (NLA) increase the risk of autism spectrum disorder (ASD) in the offspring?

**Findings:**

Using a birth cohort of more than 1.6 million children nested in nationwide health care utilization data with as long as 10 years of follow-up data, evidence of a strong association between NLA and ASD was not found after accounting for potential confounders. Meta-analysis of all recently published data yielded similar results.

**Meaning:**

While a small increase in risk cannot be excluded, given the possibility of some residual confounding, these findings do not support the notion that NLA is associated with an increased risk of ASD.

## Teaser

This cohort study evaluates whether neuraxial labor analgesia is associated with increased risk of autism spectrum disorder in the offspring.

## Introduction

Autism spectrum disorder (ASD) is a complex neurodevelopmental disorder that affects social skills, communication, and behavior control, encompassing a variety of symptoms and severity levels. While its etiology remains unknown, there is some evidence suggesting that—apart from its inheritable component^1^—the perinatal period is critical for developing ASD.^2^ A recently published cohort study including more than 147 000 singleton children, of whom approximately 74% were delivered after epidural analgesia, reported a 37% increase in risk of having a child with ASD among those with epidural analgesia exposure during labor.^3^ It was hypothesized that epidural analgesics could alter the normal course of brain maturation, potentially through placental transfer of local anesthetics.^4^

Given that epidural labor analgesia—and more generally, neuraxial labor analgesia (NLA)—is the widely accepted standard for effectively providing pain relief throughout labor, with most women in the United States using this method,^5^ the finding of an increased risk of ASD garnered substantial attention. Five US medical societies (together representing more than 100 000 physicians) as well as the British Royal College of Anaesthetists expressed strong concern that risk estimates might be biased, as important information on pregnancy and delivery complications, which are known to increase the risk of ASD and are also associated with an increased rate of epidurals, was not accounted for.^6,7^

Subsequently, 3 additional studies have examined this association.^3,8,9,10^ All reported risk estimates that were much lower than the previously observed 37% increase in risk. As these conflicting results may cause confusion and unease in pregnant women and their health care practitioners regarding the use of labor epidurals, it is crucial to obtain and communicate as much reliable information as possible about the safety of NLA and to summarize all available evidence.

Using information from 2 large population-based health care utilization databases that together contain information on women with both public and private insurance in the United States and that have rich information on potential confounders, along with a validated algorithm to identify ASD, we evaluated the association between NLA exposure and the risk of ASD in the offspring. Furthermore, we conducted a meta-analysis of all published studies focusing on this association to evaluate the totality of scientific evidence.

## Methods

### Data Source and Study Cohort

**Figure 1.**
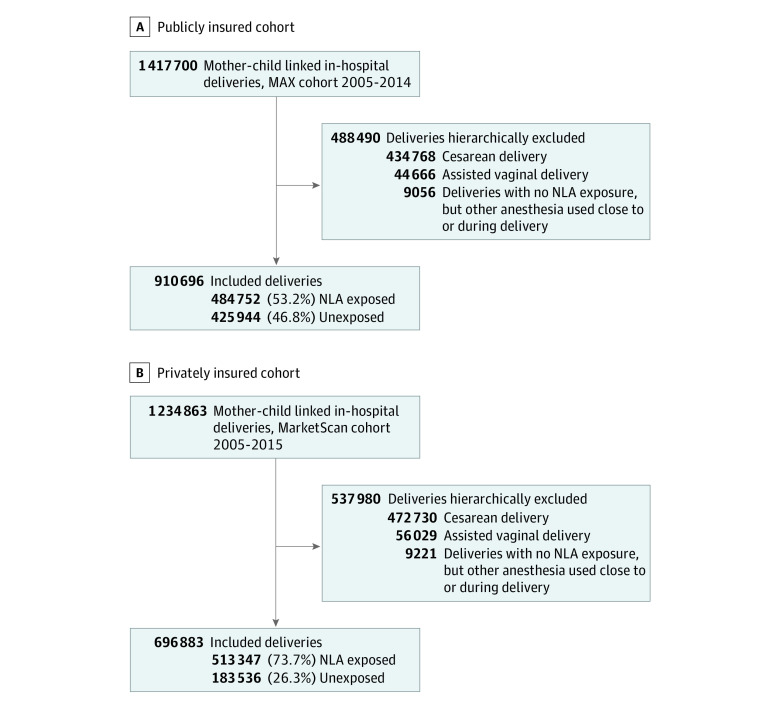
Cohort Selection for Mother-Child Dyads in the Medicaid Analytic eXtract (MAX), 2005 to 2014, and the IBM Health MarketScan Research Database, 2005 to 2015 NLA indicates neuraxial labor analgesia.

We conducted a cohort study of mothers with public and private insurance linked to their liveborn children nested in the Medicaid Analytic eXtract (MAX) from 2005 to 2014 (the most recent years available at the time of study conduct) and the IBM Health MarketScan Research Database (MarketScan) from 2005 to 2015. The MAX database consists of health care claims from the nationwide Medicaid (ie, publicly insured) populations and presents a racially diverse and relatively young population. The MarketScan database is the largest nationwide data set in the United States that is based on commercial health insurance claims. Both data sources provide rich patient-level information on demographic characteristics, insurance enrollment, outpatient medication dispensing, outpatient and emergency department visits, and hospitalizations as well as their accompanying diagnoses and procedures. For both cohorts, we have previously developed an algorithm to link mothers to their liveborn children based on state, insurance case number (to identify family units), and date of delivery.^11^ As Medicaid currently covers approximately 50% of births throughout the United States^12^ and commercial insurers cover most of the balance, these 2 cohorts together are expected to be highly representative of the nationwide obstetric population. Mothers were required to be insured from 3 months before pregnancy until 1 month after delivery to ensure complete covariate ascertainment. Start and length of pregnancy was estimated using a previously validated algorithm based on diagnostic codes for preterm birth.^13^ Children were followed up until ASD diagnosis, end of insurance enrollment, or end of the study period, whichever came first. Cesarean deliveries and assisted vaginal deliveries (ie, births performed with a forceps or vacuum device) were excluded from the main analyses but were included in sensitivity analyses (details appear in Statistical Analysis section). The exclusion and inclusion criteria are described in Figure 1.

The use of the deidentified databases for research was approved by the institutional review board at Brigham and Women’s Hospital, which waived the need for informed consent. This report follows the Strengthening the Reporting of Observational Studies in Epidemiology (STROBE) reporting guideline for observational studies.

### NLA Exposure

Exposure was based on the presence of any of the following codes indicating NLA: *International Classification of Diseases, Ninth Revision* (*ICD-9*), procedure codes 03.90 and 03.91 or *Current Procedural Terminology* (*CPT*) codes 01967, 62319, and 00955. The reference group included all women who neither had a code indicating NLA nor a code indicating other types of anesthesia used close to or during delivery (*CPT* codes 01960, anesthesia for vaginal delivery only, and 99140, anesthesia complicated by emergency conditions).

### ASD

Children with ASD were identified based on the presence of at least 2 medical encounters with a documented diagnosis of ASD (*ICD-9* codes 299.x, pervasive developmental disorders, excluding 299.1x, childhood disintegrative disorder) at 1 year or older. This algorithm has been shown to identify the outcome of ASD with a high positive predictive value based on medical record review (94% [95% CI, 83%-99%]).^14^

### Covariates

We considered a broad range of potential confounders or proxies for confounders that included demographic factors (calendar year of delivery, US state of residence, maternal age, race and ethnicity [available in MAX only]), obstetrical and labor complications (eg, obstructed/long labor, neonatal hypoxia/asphyxia, neonatal intensive care unit admission, preterm birth, preeclampsia, and multiple gestation), maternal comorbidities (eg, bipolar disorder, maternal ASD, pregestational diabetes, pregestational hypertension), prenatal medication exposure (eg, benzodiazepines, other hypnotics, antidepressants, prescription opioids), and county-level information on socioeconomic status measures (proximity to metropolitan area, unemployment rate, poverty rate, and education) (available in MAX only). For a detailed description on the individual variables and assessment periods, please refer to the eTable in the Supplement.

### Statistical Analysis

Analyses were conducted separately for the publicly and privately insured cohorts. Hazard ratios (HRs) were combined using fixed-effects meta-analysis.^15^

Balance with respect to patient characteristics between women exposed to NLA and the unexposed reference group was assessed using standardized differences estimated with the following equation: 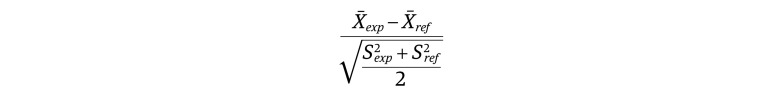
where *X̄* represents the sample mean and *s*^2^ the sample variance of the covariate in the exposed (*exp*) and the reference group (*ref*) (eTable in the Supplement). An absolute standardized difference greater than 0.1 was considered evidence of imbalance.^16^ We created crude cumulative incidence curves with 95% CIs and estimated mean age and 95% CI at first diagnosis stratified by exposure using information from Kaplan-Meier analyses.

Unadjusted and adjusted HRs and 95% CIs were estimated using Cox proportional hazards regression. Adjustment for potential confounders or proxies for confounders was done using propensity score (PS) fine stratification^17^: after trimming observations from nonoverlapping regions of the PS distribution, 50 equally sized strata were created based on the PS distribution in the exposed group. In the outcome models, unexposed observations were then weighted using the distribution of the women in the exposed group among PS strata. We accounted for covariates in a stepwise manner with increasing levels of confounding control. In model 1, we controlled for socioeconomic and demographic factors, maternal comorbidities, pregnancy conditions, and prenatal medication exposures. In model 2, we additionally accounted for labor complications and related conditions. These included variables could be both confounders or proxies for confounders (eg, affect the outcome by causing neuronal injury to the fetus and affect exposure given that women with certain conditions and labor complications might be more likely to request NLA) as well as mediators on the causal path from NLA to ASD. If they are mediators, adjustment would result in an estimate of the direct effect of NLA on ASD rather than the total effect. Thus, to account for both scenarios, analyses were conducted with and without adjustment for these conditions.

In confirmatory analyses, to account for potential residual confounding, we used high-dimensional PS analyses to identify 200 empirically defined covariates in addition to the prespecified variables included in level 2. Here again, 2 models were created: model 3 included empirical variables assessed from 3 months prior to pregnancy until the day before delivery, and model 4 included variables assessed from 3 months before pregnancy to 1 month after delivery to increase the capture of potential risk factors for ASD occurring or being recorded around the time of or shortly after delivery, similar to the variables included in model 2.

We excluded cesarean deliveries from the main analyses for several reasons. First, most cesarean deliveries in the United States are performed using neuraxial anesthesia, raising issues around defining a relevant comparator group (ie, there are virtually no unexposed pregnancies among cesarean deliveries). Second, the brief duration of exposure to local anesthetics in the context of cesarean delivery would not be expected to confer the same risk as what has been posited for labor epidurals. Third, cesarean deliveries might directly or indirectly affect the neurological development in the offspring through other pathways, such as altered microbiota or prenatal complications that result in these interventions.^2,18,19^ Finally, the study by Qiu and colleagues^3^ that reported on an increase in risk associated with NLA also did not include cesarean deliveries, and our goal was to assess whether we could replicate their findings. Nevertheless, because cesarean deliveries represent such a large proportion of deliveries in the United States and are generally done under regional anesthesia, we conducted a sensitivity analysis including cesarean deliveries and other operative deliveries.

Lastly, we conducted a literature review and meta-analysis for published studies focusing on the association of epidural/neuraxial labor analgesia with risk of ASD. We searched MEDLINE via PubMed for terms ((*epidural[Title]*) OR (*neuraxial[Title]*) OR (*analgesia[Title]*) OR (*anesthesia[Title]*)) AND (*Autism[Title]*). Articles were included if they reported HRs for epidural/NLA exposure and ASD. Animal studies, basic science research, case reports, case series, editorials, letters to the editor, and commentaries were excluded. We performed a fixed-effects meta-analysis, using the highest-level adjusted estimates reported in each of the studies, quantified between-study variability using the χ^2^ test of heterogeneity, and summarized results in a forest plot.

Analyses were conducted using SAS version 9.4 (SAS Institute, Inc). Statistical significance was set at the 5% level, and all tests were 2-tailed.

## Results

### Cohort Characteristics

The publicly insured cohort consisted of 910 696 pregnancies (mean [SD] maternal age, 24.3 [5.7] years; 286 025 [31.4%] Black mothers; 374 282 [41.1%] White mothers), of which 484 752 (53.2%) were exposed to NLA. The privately insured cohort included 696 883 pregnancies (mean [SD] maternal age, 31.0 [4.5] years; race and ethnicity data not available), with 513 347 (73.7%) being exposed. When comparing those with vs without NLA exposure within cohorts, the strongest imbalances observed in the publicly insured cohort were that women with NLA exposure were more likely to have delivered in the more recent years; to be younger; to be White; to be from the Midwest or South; to have active smoking; to be diagnosed with hyperemesis/vomiting in pregnancy; to be exposed to prescription opioids, antidepressants, or suspected teratogens; to have more outpatient visits; and to have obstructed/long labor (Table; eTable in the Supplement). No substantial differences were observed for other characteristics. Among women with private insurance, those exposed to NLA were more likely to be from the South than from the West and to be exposed to prescription opioids, but other covariates were generally balanced in this population.

**Table.** Selected Cohort Characteristics of Pregnancies With and Without Exposure to Neuraxial Labor Analgesia During Delivery, by Insurance Type

Characteristic	Publicly insured cohort (MAX 2005-2014)			Privately insured cohort (MarketScan 2005-2015)		
	Deliveries, No. (%)		Standardized difference^a^	Deliveries, No. (%)		Standardized difference^a^
	Exposed (n = 484 752)	Unexposed (n = 425 944)		Exposed (n = 513 347)	Unexposed (n = 183 536)	
Demographic factors						
Maternal age, mean (SD), y	23.9 (5.6)	24.8 (5.9)	−0.16	31.5 (4.5)	31.8 (4.6)	−0.07
Calendar year of delivery						
≤2007	119 466 (24.6)	141 790 (33.3)	−0.19	90 389 (17.6)	36 482 (19.9)	−0.06
2008-2011	220 428 (45.5)	184 214 (43.3)	0.04	223 629 (43.6)	78 485 (42.8)	0.02
≥2012	144 858 (29.9)	99 940 (23.5)	0.15	199 329 (38.8)	68 569 (37.4)	0.03
US region of residence						
Northeast	88 245 (18.2)	102 501 (24.1)	−0.14	77 341 (15.1)	33 683 (18.4)	−0.09
Midwest	174 555 (36.0)	119 891 (28.2)	0.17	139 866 (27.3)	50 772 (27.7)	−0.01
South	138 907 (28.7)	95 686 (22.5)	0.14	206 961 (40.3)	55 080 (30.0)	0.22
West	83 045 (17.1)	107 866 (25.3)	−0.20	83 205 (16.2)	41 623 (22.7)	−0.16
Race and ethnicity^b^						
Asian	12 321 (2.5)	25 370 (6.0)	−0.17	NA^c^	NA^c^	NA^c^
Black/African American	150 467 (31.0)	135 558 (31.8)	−0.02	NA^c^	NA^c^	NA^c^
Hispanic/Latino^d^	52 460 (10.8)	68 826 (16.2)	−0.16	NA^c^	NA^c^	NA^c^
Unknown/other^e^	44 051 (9.1)	47 361 (11.1)	−0.07	NA^c^	NA^c^	NA^c^
White	225 453 (46.5)	148 829 (34.9)	0.24	NA^c^	NA^c^	NA^c^
Obstetrical and labor complications						
Obstructed/long labor	21 715 (4.5)	11 327 (2.7)	0.10	22 316 (4.4)	4981 (2.7)	0.09
Maternal pyrexia or infection during labor	4126 (0.9)	1621 (0.4)	0.06	5168 (1.0)	617 (0.3)	0.08
Chorioamnionitis	15 249 (3.2)	9829 (2.3)	0.05	11 538 (2.3)	2622 (1.4)	0.06
Neonatal hypoxia/asphyxia	3515 (0.7)	2877 (0.7)	0.01	3657 (0.7)	1031 (0.6)	0.02
NICU admission	26 966 (5.6)	19 930 (4.7)	0.04	27 045 (5.3)	9639 (5.3)	0.00
Gestational age at birth, completed weeks^f^						
≤28	963 (0.2)	2451 (0.6)	−0.06	813 (0.2)	801 (0.4)	−0.05
>29-32	2373 (0.5)	3474 (0.8)	−0.04	1092 (0.2)	955 (0.5)	−0.05
>33-36	37 068 (7.7)	34 797 (8.2)	−0.02	29 548 (5.8)	11 558 (6.3)	−0.02
≥37	444 348 (91.7)	385 222 (90.4)	0.04	481 894 (93.9)	170 222 (92.8)	0.05
Preeclampsia	23 448 (4.8)	14 761 (3.5)	0.07	23 041 (4.5)	5925 (3.2)	0.07
Multiple gestation	3205 (0.7)	2000 (0.5)	0.03	4134 (0.8)	1073 (0.6)	0.03
Maternal comorbidities						
Bipolar disorder	16 262 (3.4)	9701 (2.3)	0.07	2473 (0.5)	763 (0.4)	0.01
Maternal autism spectrum disorder	106 (0.0)	156 (0.0)	−0.01	27 (0.0)	12 (0.0)	0.00
Pregestational diabetes	24 338 (5.0)	19 998 (4.7)	0.02	34 049 (6.6)	11 273 (6.1)	0.02
Pregestational hypertension	21 953 (4.5)	14 121 (3.3)	0.06	23 744 (4.6)	6066 (3.3)	0.07
Maternal medication exposure						
Benzodiazepines	21 927 (4.5)	12 181 (2.9)	0.09	19 160 (3.7)	4286 (2.3)	0.08
Other hypnotics	41 726 (8.6)	28 553 (6.7)	0.07	20 660 (4.0)	4826 (2.6)	0.08
Antidepressants	60 681 (12.5)	38 141 (9.0)	0.12	44 017 (8.6)	11 329 (6.2)	0.09
Prescription opioids	164 620 (34.0)	112 079 (26.3)	0.17	87 814 (17.1)	24 257 (13.2)	0.11

Abbreviations: MarketScan, IBM Health MarketScan Research Database; MAX, Medicaid Analytic eXtract; NA, not applicable; NICU, neonatal intensive care unit.

^a^ Standardized differences were calculated as described in the text.

^b^ Race and ethnicity was determined using information submitted to the Centers for Medicare & Medicaid Services by individual states, which was based on information that had been collected and coded from Medicaid applications.

^c^ Information not available in MarketScan.

^d^ No race information available.

^e^ Unknown or other includes the following racial and ethnic groups: American Indian or Alaska Native, Native Hawaiian or other Pacific Islander, Hispanic or Latino, and 1 or more races, more than 1 race, and unknown.

^f^ Estimated using a previously validated algorithm based on diagnostic codes for preterm birth.^13^

### Mean Age, Cumulative Incidence, and HRs of ASD

**Figure 2.**
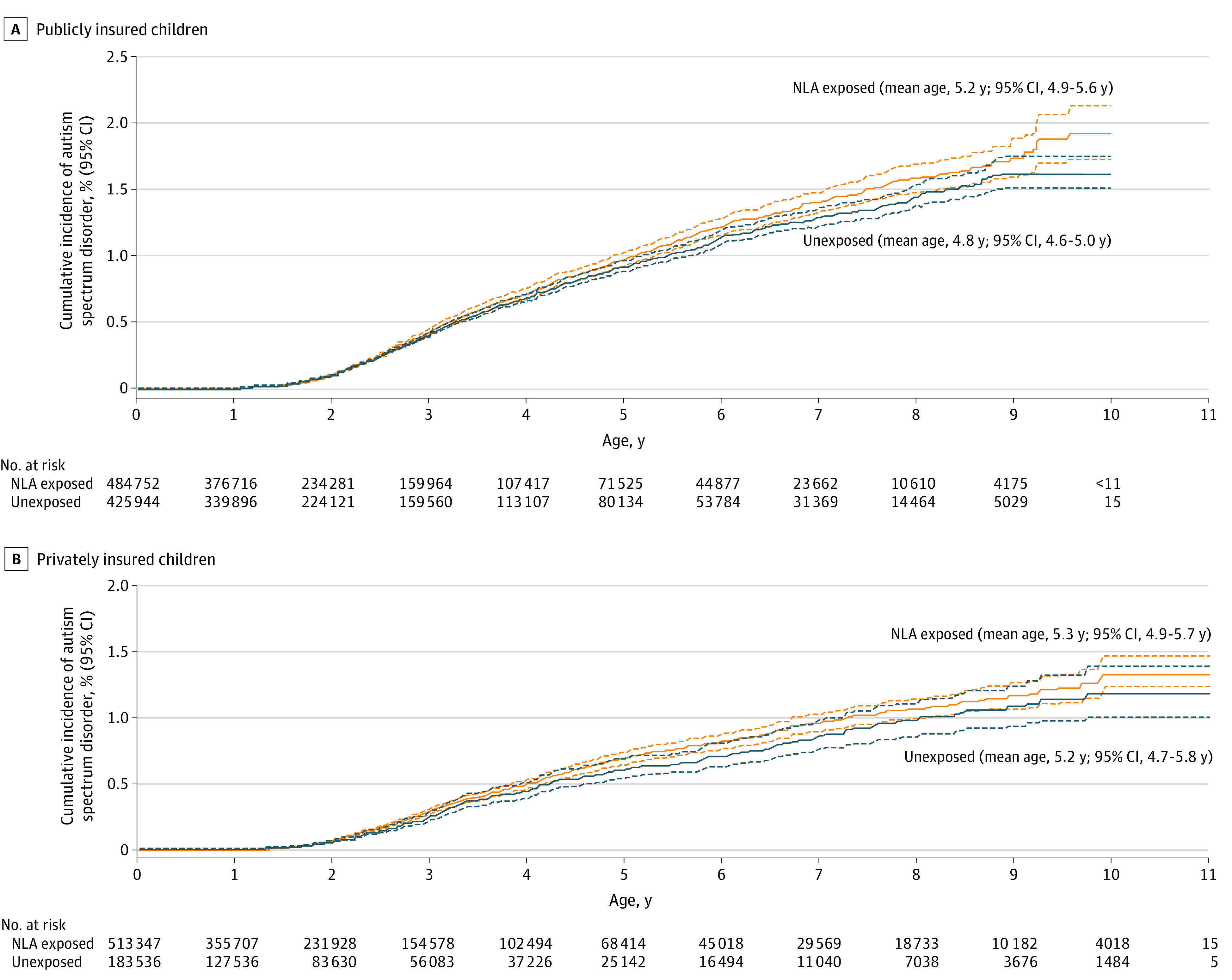
Cumulative Incidence and Mean Age at Diagnosis of Autism Spectrum Disorder in Children Whose Mothers Were Exposed to Neuraxial Labor Analgesia (NLA) vs Children Without Exposure, by Insurance Type Solid lines represent cumulative incidences, dashed lines represent 95% CIs. Cell sizes less than 11 in the publicly insured cohort were suppressed in accord with the Centers for Medicare & Medicaid Services cell size suppression policy.

**Figure 3.**
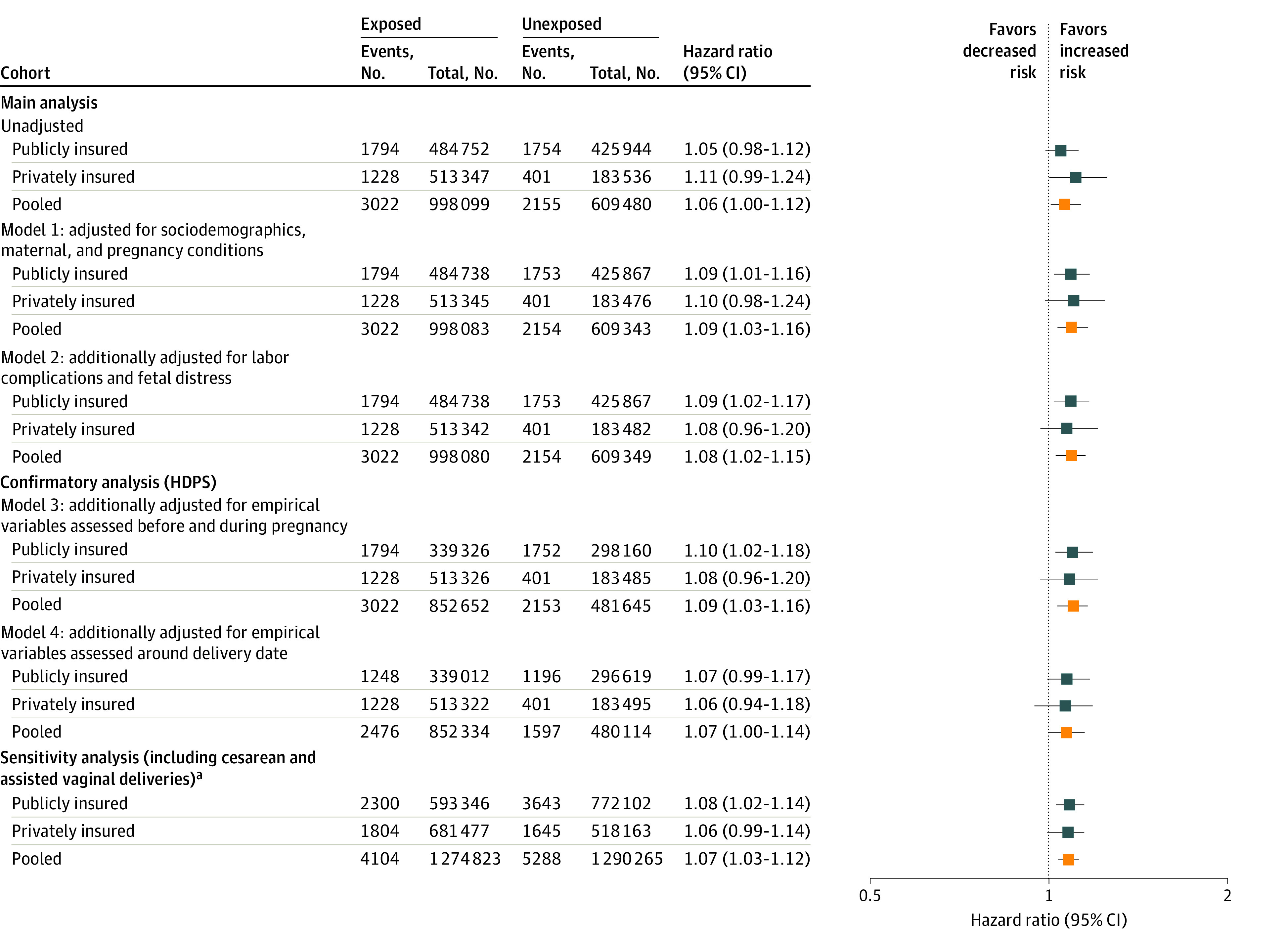
Hazard Ratios of Autism Spectrum Disorder in Children Whose Mothers Were Exposed to Neuraxial Labor Analgesia vs Those Without Exposure Unadjusted and adjusted results are shown for each cohort separately and after pooling. Adjustment using propensity score fine stratification was conducted in a stepwise manner to illustrate the consequences of gradually increased confounding control. Pooled hazard ratios were estimated using fixed-effects meta-analysis. Model 1 accounts for socioeconomic and demographic factors, maternal comorbidities, pregnancy conditions, and prenatal medication exposures; model 2, all variables included in model 1, labor complications, and fetal distress; model 3, all variables included in model 2 and 200 empirical variables assessed from 3 months before pregnancy to 1 day before delivery; model 4, all variables included in model 2 and 200 empirical variables assessed from 3 months before pregnancy to 30 days after delivery. HDPS indicates high-dimensional propensity score. ^a^Hazard ratios from sensitivity analyses represent results from model 2–adjusted analyses with additional inclusion of mode of delivery (vaginal delivery, assisted vaginal delivery, cesarean delivery) into the propensity score model. Model 3 and 4 analyses were not repeated in this extended cohort, as HDPS did not substantially change results from the main analyses.

The mean age at first ASD diagnosis (accounting for right-censoring through Kaplan-Meier analysis) of the 3548 ASD cases in the publicly insured cohort was 5.2 (95% CI, 4.9-5.6) years among those exposed to NLA (1794 cases) and 4.8 (95% CI, 4.6-5.0) years among those not exposed to NLA (1754 cases) (Figure 2). Respective mean ages among the 1629 children with private insurance and ASD diagnosis were 5.3 years (95% CI, 4.9-5.7 years; 1228 cases) and 5.2 years (95% CI, 4.7-5.8 years; 401 cases). Cumulative incidence of ASD by age 10 years was 1.93% (95% CI, 1.73%-2.13%) among children with NLA exposure vs 1.64% (95% CI, 1.51%-1.76%) among those without in the publicly insured cohort, and 1.33% (95% CI, 1.19%-1.46%) among children with NLA exposure vs 1.19% (95% CI, 0.99%-1.38%) among those without in the privately insured cohort, corresponding to an unadjusted pooled HR of 1.06 (95% CI, 1.00-1.12) (Figure 3). When adjusting for potential confounders, results were consistent with the unadjusted analysis. Using the highest level of adjustment based on predefined variables (model 2), the HR was 1.09 (95% CI, 1.02-1.17) for the publicly insured cohort and 1.08 (95% CI, 0.96-1.20) for the privately insured cohort, resulting in a pooled HR of 1.08 (95% CI, 1.02-1.15). Using the highest level of adjustment in the confirmatory analyses based on both predefined and empirically identified variables (model 4), the HRs were 1.07 (95% CI, 0.99-1.17) and 1.06 (95% CI, 0.94-1.18) for the publicly and privately insured cohorts respectively, corresponding to a pooled HR of 1.07 (95% CI, 1.00-1.14).

### Sensitivity Analysis

Including cesarean deliveries and assisted vaginal deliveries substantially increased the cohort size to 1 365 461 in the publicly insured cohort and 1 199 813 in the privately insured cohort. Adjusted results were very similar to those from the main analyses (pooled HR for model 2 analysis, 1.07; 95% CI, 1.03-1.12) (Figure 3).

### Meta-analysis With Previous Studies

**Figure 4.**
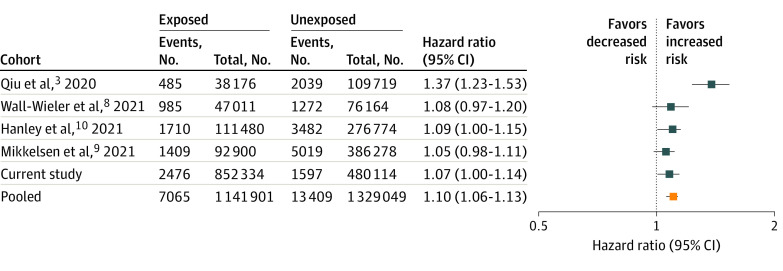
Meta-analysis of Published Studies on the Association of Epidural/Neuraxial Analgesia During Labor/Delivery With the Risk of Autism Spectrum Disorder in the Offspring Highest-level adjusted estimates reported in each study are shown, including results from model 4 of the current study. Pooled hazard ratios were estimated using fixed-effects meta-analysis.

The PubMed search using our defined search criteria initially yielded 37 citations, of which 15 were directly related to this research question, and 4 presented original data.^3,8,9,10^ All 4 studies were included in the meta-analysis and were pooled with the results from our analyses. Results from the meta-analysis yielded a pooled adjusted HR of 1.10 (95% CI, 1.06-1.13) (Figure 4) with a *P* value from the χ^2^ test of .001, suggesting between-study heterogeneity. Excluding the study by Qiu et al^3^ from the meta-analysis resulted in a similar, if slightly attenuated, estimate (pooled adjusted HR, 1.07; 95% CI, 1.04-1.11); however, the *P* value (.82) suggested very low heterogeneity.

## Discussion

Using 2 nationwide mother-child linked birth cohorts that together included more than 1.6 million children born vaginally, we did not find evidence of a strong association between NLA exposure and ASD risk. Estimates were consistent when expanding the cohort to include children born via cesarean deliveries or assisted vaginal deliveries. Results were also consistent when meta-analyzing all data published so far, with the upper bound of the 95% CI excluding a more than 13% increase in risk. Therefore, the combined evidence from these observational studies does not indicate a strong association between NLA and ASD.

The first large observational study (by Qiu et al^3^) to evaluate such an association reported a 37% increase in risk with epidural analgesia during labor. However, the 3 cohort studies that were published shortly thereafter all reported adjusted risk estimates similar to those observed in our data, with HRs ranging from 1.05 to 1.09 and the highest upper confidence limit at 1.20.^8,9,10^ While the estimate from the meta-analysis shifted only slightly toward the null (from 1.10 to 1.07) when excluding the study by Qiu and colleagues,^3^ statistical testing no longer pointed toward heterogeneity between studies, confirming that the study by Qiu et al^3^ is indeed substantially different from the others. The explanation for the increase observed in that first study is not clear; residual confounding particular to that study population (eg, by labor complications, maternal mental health issues, or socioeconomic characteristics, factors that have been insufficiently accounted for in the analyses) is a possibility. The biological plausibility of an association between epidural labor analgesia and ASD is questionable because the diluted local anesthetics and opioids are infused into the mother’s epidural space at such low doses that a fetal neurotoxic effect appears very unlikely.^20^

### Strengths and Limitations

Strengths of this study include using very large cohorts of mother-child dyads with both public and private insurance as well as continuous follow-up of the children to age 10 years. To our knowledge, these cohorts together are more than 3 times larger (5 times when including cesarean deliveries and assisted vaginal deliveries) than the biggest cohort study published to date focusing on the association of interest and are expected to be broadly representative of the US obstetric population. The large size coupled with rich information on a broad range of covariates and the use of a validated outcome definition allowed for precise risk estimation with robust confounding adjustment.

This study also has limitations. As both outcome and exposure are based on the presence of specific diagnostic and procedure codes, we need to equate absence of a claim to absence of a condition or treatment. While our algorithm to identify ASD has yielded a high positive predictive value,^21^ ASD cases that were either not recorded or did not meet our definition were missed, thus resulting in a potential underestimation of the true number of children with ASD. However, the ASD incidences observed in our cohorts align well with autism prevalence figures reported by the US Centers for Disease Control and Prevention.^22,23,24^ Furthermore, we opted for a highly specific outcome definition, which will result in nonbiased or minimally biased HRs assuming nondifferential sensitivity. As information in our databases is based on claims used for billing, health care professionals have a strong incentive to provide complete information and are regularly audited to ensure they do not report diagnoses or procedures that do not exist, which would indicate fraud. Because of this, coding of NLA is expected to be accurate. Moreover, as a prerequisite of this study, we explored the feasibility of using the selected *ICD-9* and *CPT* codes by reviewing maternal claims profiles and comparing our estimated NLA prevalence to existing literature^5,25^ and found that these codes seemed to adequately capture NLA use. Residual confounding is always a concern in nonrandomized studies, and the small increase in risk we observed is likely attributable to residual confounding. We were not able to address a potential dose-response association, as our data do not contain information on NLA duration and dosage. Lastly, we restricted our main analyses to vaginal deliveries because our data did not differentiate between neuraxial analgesia prior to or during cesarean delivery nor did they allow us to adequately distinguish different anesthesia methods used during cesarean delivery. Nevertheless, this restriction is not expected to result in selection bias because NLA has been shown not to increase the risk of cesarean deliveries.^26^ Furthermore, when including cesarean delivery and assisted vaginal deliveries, adjusted results were very consistent with those from our main analyses.

## Conclusions

Overall, findings from our study as well as the results from the meta-analysis do not suggest a strong association of NLA use with risk of ASD. While we cannot exclude a small increase in risk, given the observational nature of these types of studies, this small elevation can easily be explained by a small amount of residual confounding related to differences in underlying maternal, obstetric/labor, and genetic factors. Given that childbirth is typically the most painful moment in a woman’s life, maternal pain can lead to severe consequences (eg, long-term psychological suffering),^27,28^ and NLA is the widely accepted standard for effective and safe labor analgesia used by most pregnant women in the United States, these findings are important and reassuring for both pregnant women and their health care practitioners when considering options for labor pain relief.

## Notes

Supplement. **eTable. **Cohort Characteristics of Pregnancies With and Without Exposure to Neuraxial Labor Analgesia During Delivery, Separately for Public and Private Insurance Cohort
eReferences.
