# Supplementary material for: Association of Neuraxial Labor Analgesia for Vaginal Childbirth With Risk of Autism Spectrum Disorder
Source: JAMA Netw Open. 2021 Dec 22;4(12):e2140458. doi: 10.1001/jamanetworkopen.2021.40458 (PMC8696569; doi:10.1001/jamanetworkopen.2021.40458)
Supplement: Supplement. — eTable. Cohort Characteristics of Pregnancies With and Without Exposure to Neuraxial Labor Analgesia During Delivery, Separately for Public and Private Insurance Cohort eReferences. [file jamanetwopen-e2140458-s001.pdf]

Supplemental Online Content

Straub L, Huybrechts KF, Mogun H, Bateman BT. Association of neuraxial labor analgesia for vaginal childbirth with risk of autism spectrum disorder. *JAMA Netw Open.* 2021;4(12):e2140458. doi:10.1001/jamanetworkopen.2021.40458

eTable. Cohort Characteristics of Pregnancies With and Without Exposure to Neuraxial Labor Analgesia During Delivery, Separately for Public and Private Insurance Cohort
eReferences.

This supplemental material has been provided by the authors to give readers additional information about their work.

© 2021 Straub L et al. *JAMA Network Open.*

eTable. Cohort Characteristics of Pregnancies With and Without Exposure to Neuraxial Labor Analgesia During Delivery, Separately for Public and Private Insurance Cohort

Data are Number (%) Unless Stated Otherwise.

	Publicly Insured Cohort (MAX 2005-2014)					Privately Insured Cohort (MarketScan 2005-2015)					Included in Outcome Model[a]
	Exposed		Unexposed		Std.Diff	Exposed		Unexposed		Std.Diff	
	N/Mean	%/SD	N/Mean	%/SD		N/Mean	%/SD	N/Mean	%/SD		
Total Number of Deliveries	484,752		425,944			513,347		183,536			
**Demographic Factors**											
Maternal Age (in Years) (Mean (SD))	23.9	5.6	24.8	5.9	-0.16	31.5	4.5	31.8	4.6	-0.07	
Calendar Year of Delivery:											model 1 (individual years)
<=2007	119,466	24.6	141,790	33.3	-0.19	90,389	17.6	36,482	19.9	-0.06	
2008-2011	220,428	45.5	184,214	43.3	0.04	223,629	43.6	78,485	42.8	0.02	
>=2012	144,858	29.9	99,940	23.5	0.15	199,329	38.8	68,569	37.4	0.03	
US Region of Residence:											model 1 (individual states)
Northeast	88,245	18.2	102,501	24.1	-0.14	77,341	15.1	33,683	18.4	-0.09	
Midwest	174,555	36.0	119,891	28.2	0.17	139,866	27.3	50,772	27.7	-0.01	
South	138,907	28.7	95,686	22.5	0.14	206,961	40.3	55,080	30.0	0.22	
West	83,045	17.1	107,866	25.3	-0.20	83,205	16.2	41,623	22.7	-0.16	
Race/Ethnicity[b]:											model 1
Asian/Other Pacific Islander	12,321	2.5	25,370	6.0	-0.17	[c]	[c]	[c]	[c]	[c]	
Black/African American	150,467	31.0	135,558	31.8	-0.02	[c]	[c]	[c]	[c]	[c]	
Hispanic/Latino	52,460	10.8	68,826	16.2	-0.16	[c]	[c]	[c]	[c]	[c]	
Unknown/Other	44,051	9.1	47,361	11.1	-0.07	[c]	[c]	[c]	[c]	[c]	
White	225,453	46.5	148,829	34.9	0.24	[c]	[c]	[c]	[c]	[c]	
**Obstetrical & Labor Comlications**											
Hyperemesis/Nasea & Vomiting in Pregnancy	102,249	21.1	67,764	15.9	0.13	42,899	8.4	12,538	6.8	0.06	model 1
Obstetric Comorbidity Score[d]:											model 1
0	313,836	64.7	289,932	68.1	-0.07	286,198	55.8	104,354	56.9	-0.02	
1	78,614	16.2	66,874	15.7	0.01	120,968	23.6	44,641	24.3	-0.02	
2	53,682	11.1	41,747	9.8	0.04	62,603	12.2	21,238	11.6	0.02	
>=3	38,620	8.0	27,391	6.4	0.06	43,578	8.5	13,303	7.3	0.05	
Obstructed/Long Labor	21,715	4.5	11,327	2.7	0.10	22,316	4.4	4,981	2.7	0.09	model 2

© 2021 Straub L et al. *JAMA Network Open.*

	Publicly Insured Cohort (MAX 2005-2014)					Privately Insured Cohort (MarketScan 2005-2015)					Included in Outcome Model[a]
	Exposed		Unexposed		Std.Diff	Exposed		Unexposed		Std.Diff	
	N/Mean	%/SD	N/Mean	%/SD		N/Mean	%/SD	N/Mean	%/SD		
Delayed Delivery After Rupture of Membrane	7,069	1.5	5,745	1.4	0.01	5,865	1.1	2,215	1.2	-0.01	model 2
Other Labor Complications[e]	276,360	57.0	239,723	56.3	0.01	365,116	71.1	131,238	71.5	-0.01	model 2
Fetal Malposition/Malpresentation/Disproportion	15,212	3.1	13,079	3.1	0.00	16,137	3.1	5,177	2.8	0.02	model 2
Labor Dystocia	12,635	2.6	8,096	1.9	0.05	11,193	2.2	3,862	2.1	0.01	model 2
Induction of Labor[f]	2,073	0.4	1,312	0.3	0.02	1,745	0.3	383	0.2	0.03	model 2
Maternal Pyrexia or Infection During Labor[g]	4,126	0.9	1,621	0.4	0.06	5,168	1.0	617	0.3	0.08	
Chorioamnionitis[g]	15,249	3.2	9,829	2.3	0.05	11,538	2.3	2,622	1.4	0.06	
Neonatal Hypoxia/Asphyxia	3,515	0.7	2,877	0.7	0.01	3,657	0.7	1,031	0.6	0.02	model 2
Length of Hospitalization After Delivery:											model 2
0-3 Days	340,291	70.2	325,200	76.4	-0.14	377,558	73.6	150,790	82.2	-0.21	
4-5 Days	118,755	24.5	76,936	18.1	0.16	122,930	24.0	26,557	14.5	0.24	
6-10 Days	14,033	2.9	11,426	2.7	0.01	8,878	1.7	3,575	2.0	-0.02	
>=11 Days	11,673	2.4	12,382	2.9	-0.03	3,981	0.8	2,614	1.4	-0.06	
NICU Admission	26,966	5.6	19,930	4.7	0.04	27,045	5.3	9,639	5.3	0.00	model 2
Small for Gestational Age	17,835	3.7	12,531	2.9	0.04	14,599	2.8	4,492	2.5	0.02	model 2
Low Birth Weight	845	0.2	819	0.2	0.00	461	0.1	171	0.1	0.00	model 2
Gestational Age at Birth[h]:											model 2
<=28 Weeks	963	0.2	2,451	0.6	-0.06	813	0.2	801	0.4	-0.05	
>29-32 Weeks	2,373	0.5	3,474	0.8	-0.04	1,092	0.2	955	0.5	-0.05	
>33-36 Weeks	37,068	7.7	34,797	8.2	-0.02	29,548	5.8	11,558	6.3	-0.02	
>=37 Weeks	444,348	91.7	385,222	90.4	0.04	481,894	93.9	170,222	92.8	0.05	
Preeclampsia	23,448	4.8	14,761	3.5	0.07	23,041	4.5	5,925	3.2	0.07	model 2
Multiple Gestation	3,205	0.7	2,000	0.5	0.03	4,134	0.8	1,073	0.6	0.03	model 1
Parity	334,648	69.0	316,957	74.4	-0.12	[c]	[c]	[c]	[c]	[c]	model 1
**Healthcare Utilization**											
Number of Outpatient Visits in Quartiles:											model 1
1	89451	18.45	126376	29.67	-0.26	115612	22.52	52584	28.65	-0.14	
2	109986	22.69	99556	23.37	-0.02	158786	30.93	55833	30.42	0.01	
3	142749	29.45	109767	25.77	0.08	119004	23.18	39051	21.28	0.05	
4	142566	29.41	90245	21.19	0.19	119945	23.37	36068	19.65	0.09	
Number of Hospitalizations:											model 1
0	273743	56.47	280180	65.78	-0.19	486873	94.84	174625	95.14	-0.01	

© 2021 Straub L et al. *JAMA Network Open.*

	Publicly Insured Cohort (MAX 2005-2014)					Privately Insured Cohort (MarketScan 2005-2015)					Included in Outcome Model[a]
	Exposed		Unexposed		Std.Diff	Exposed		Unexposed		Std.Diff	
	N/Mean	%/SD	N/Mean	%/SD		N/Mean	%/SD	N/Mean	%/SD		
1	175372	36.18	113973	26.76	0.20	19741	3.85	6843	3.73	0.01	
2-3	29579	6.1	21412	5.03	0.05	3652	0.71	1159	0.63	0.01	
>=4	6058	1.25	10379	2.44	-0.09	3081	0.6	909	0.5	0.01	
**Shared Environmental Factors**											
Tobacco Use	59,613	12.3	32,182	7.6	0.16	6,162	1.2	1,693	0.9	0.03	model 1
Substance Use Disorder/Dependence	21,692	4.5	13,589	3.2	0.07	1,050	0.2	330	0.2	0.01	model 1
Alcohol Use Disorder/Dependence	5,402	1.1	4,173	1.0	0.01	561	0.1	172	0.1	0.00	model 1
**Maternal Comorbidities**											
Depression with Psychotic Features	1,318	0.3	1,103	0.3	0.00	106	0.0	46	0.0	0.00	model 1
Bipolar Disorder	16,262	3.4	9,701	2.3	0.07	2,473	0.5	763	0.4	0.01	model 1
Schizophrenia/Schizoaffective Disorder	1,294	0.3	1,063	0.3	0.00	69	0.0	24	0.0	0.00	model 1
Other Mental Health Issues[i]	45,321	9.4	31,910	7.5	0.07	36,363	7.1	11,216	6.1	0.04	model 1
Epilepsy/Convulsion	5,915	1.2	4,033	1.0	0.03	2,166	0.4	654	0.4	0.01	model 1
Migraine/Headache	65,367	13.5	44,553	10.5	0.09	34,841	6.8	10,272	5.6	0.05	model 1
Maternal Autism Spectrum Disorder	106	0.0	156	0.0	-0.01	27	0.0	12	0.0	0.00	model 1
Maternal Attention Deficit Hyperactivity Disorder	6,369	1.3	3,122	0.7	0.06	2,780	0.5	536	0.3	0.04	model 1
Maternal History of Other Developmental Disorders[j]	1,468	0.3	1,290	0.3	0.00	78	0.0	27	0.0	0.00	model 1
Overweight/Obesity	33,023	6.8	21,862	5.1	0.07	16,098	3.1	4,725	2.6	0.03	model 1
Pre-gestational Diabetes	24,338	5.0	19,998	4.7	0.02	34,049	6.6	11,273	6.1	0.02	model 1
Gestational Diabetes	825	0.2	648	0.2	0.00	648	0.1	223	0.1	0.00	model 1
Pre-gestational Hypertension	21,953	4.5	14,121	3.3	0.06	23,744	4.6	6,066	3.3	0.07	model 1
Gestational Hypertension	20,156	4.2	11,363	2.7	0.08	22,998	4.5	5,515	3.0	0.08	model 1
Autoimmune Disorders[k]	5,400	1.1	4,053	1.0	0.02	11,815	2.3	3,746	2.0	0.02	model 1
Asthma Bronchiale	38,603	8.0	27,469	6.5	0.06	15,603	3.0	5,042	2.8	0.02	model 1
TORCH or other STDs	44,518	9.2	33,897	8.0	0.04	9,027	1.8	2,985	1.6	0.01	model 1
Group B Streptococcus Infection	40,369	8.3	25,087	5.9	0.09	10,356	2.0	3,470	1.9	0.01	model 1
Previous Cesarean Delivery	9,555	2.0	6,801	1.6	0.03	4,622	0.9	2,006	1.1	-0.02	model 1
**Maternal Medication Exposure**											
Benzodiazepines	21,927	4.5	12,181	2.9	0.09	19,160	3.7	4,286	2.3	0.08	model 1
Other Hypnotics	41,726	8.6	28,553	6.7	0.07	20,660	4.0	4,826	2.6	0.08	model 1
Barbiturates	14,375	3.0	7,667	1.8	0.08	12,498	2.4	2,726	1.5	0.07	model 1
Anxiolytics	3,975	0.8	2,031	0.5	0.04	1,572	0.3	380	0.2	0.02	model 1

© 2021 Straub L et al. *JAMA Network Open.*

	Publicly Insured Cohort (MAX 2005-2014)					Privately Insured Cohort (MarketScan 2005-2015)					Included in Outcome Model[a]
	Exposed		Unexposed		Std.Diff	Exposed		Unexposed		Std.Diff	
	N/Mean	%/SD	N/Mean	%/SD		N/Mean	%/SD	N/Mean	%/SD		
Anticonvulsants	13,909	2.9	8,759	2.1	0.05	5,780	1.1	1,516	0.8	0.03	model 1
Antidepressants	60,681	12.5	38,141	9.0	0.12	44,017	8.6	11,329	6.2	0.09	model 1
Psychostimulants	6,213	1.3	2,786	0.7	0.06	4,413	0.9	809	0.4	0.05	model 1
Antipsychotics	9,856	2.0	6,160	1.5	0.04	1,302	0.3	375	0.2	0.01	model 1
Antidiabetics	7,950	1.6	6,130	1.4	0.02	15,962	3.1	4,288	2.3	0.05	model 1
Insulin	5,925	1.2	4,683	1.1	0.01	6,122	1.2	1,916	1.0	0.01	model 1
Antihypertensives	27,717	5.7	20,169	4.7	0.04	25,604	5.0	7,283	4.0	0.05	model 1
Prescription Opioids	164,620	34.0	112,079	26.3	0.17	87,814	17.1	24,257	13.2	0.11	model 1
Treatment of Opioid Use Disorder/Overdose	3,723	0.8	1,718	0.4	0.05	340	0.1	85	0.1	0.01	model 1
Triptans	6,654	1.4	4,619	1.1	0.03	7,383	1.4	1,836	1.0	0.04	model 1
NSAIDs/Other Analgesics	113,872	23.5	94,699	22.2	0.03	39,359	7.7	10,769	5.9	0.07	model 1
Corticosteroids	96,029	19.8	74,036	17.4	0.06	99,023	19.3	29,570	16.1	0.08	model 1
Progestins	28,352	5.9	20,722	4.9	0.04	64,255	12.5	18,312	10.0	0.08	model 1
Prenatal Folate Supplements	328,098	67.7	273,419	64.2	0.07	214,226	41.7	67,550	36.8	0.10	model 1
Suspected Teratogens	97,561	20.1	67,405	15.8	0.11	58,304	11.4	17,783	9.7	0.05	model 1
Known Teratogens	2,288	0.5	1,581	0.4	0.02	2,398	0.5	724	0.4	0.01	model 1
**Socioeconomic Factors**[l]											
Proximity to Metropolitan Area:											model 1
Metropolitan	426,005	87.9	368,800	86.6	0.04	c	c	c	c	c	
Urban	49,580	10.2	48,999	11.5	-0.04	c	c	c	c	c	
Rural	9,167	1.9	8,145	1.9	0.00	c	c	c	c	c	
Unemployment Rate:											model 1
Low	127,993	26.4	102,128	24.0	0.06	c	c	c	c	c	
Low - Moderate	129,826	26.8	102,071	24.0	0.06	c	c	c	c	c	
Moderate - High	122,319	25.2	109,848	25.8	-0.01	c	c	c	c	c	
High	104,614	21.6	111,897	26.3	-0.11	c	c	c	c	c	
Poverty Rate:											model 1
Low	124,184	25.6	105,573	24.8	0.02	c	c	c	c	c	
Low - Moderate	110,182	22.7	102,364	24.0	-0.03	c	c	c	c	c	
Moderate - High	126,556	26.1	99,520	23.4	0.06	c	c	c	c	c	
High	123,830	25.6	118,487	27.8	-0.05	c	c	c	c	c	
Population % with <= High School Diploma:											model 1

© 2021 Straub L et al. *JAMA Network Open.*

	Publicly Insured Cohort (MAX 2005-2014)					Privately Insured Cohort (MarketScan 2005-2015)					Included in Outcome Model[a]
	Exposed		Unexposed		Std.Diff	Exposed		Unexposed		Std.Diff	
	N/Mean	%/SD	N/Mean	%/SD		N/Mean	%/SD	N/Mean	%/SD		
Low	125,677	25.9	104,989	24.7	0.03	c	c	c	c	c	
Low - Moderate	127,880	26.4	96,053	22.6	0.09	c	c	c	c	c	
Moderate - High	113,878	23.5	112,813	26.5	-0.07	c	c	c	c	c	
High	117,317	24.2	112,089	26.3	-0.05	c	c	c	c	c	

Abbreviations: MarketScan, IBM Health MarketScan Research Database; MAX, Medicaid Analytic eXtract; N, number; NICU, neonatal intensive care unit; SD, standard deviation; STD, sexually transmitted disease; Std.Diff, standardized difference; TORCH, acronym representing infections caused by Toxoplasma gondii, other agents, rubella, cytomegalovirus, and herpes simplex virus.

Assessment periods:

- Delivery-14 days to delivery+30 days: obstructed/long labor, delayed delivery after rupture of membrane, other labor complications, fetal malposition/malpresentation/disproportion, labor dystocia, induction of labor, maternal pyrexia or infection during labor, chorioamnionitis
- Delivery to delivery+30 days: neonatal hypoxia/asphyxia, length of hospitalization after delivery, nicu, small for gestational age, low birth weight, gestational age at birth
- Last menstrual period+140 days to delivery+30 days: preeclampsia
- Last menstrual period to delivery+60 days: multiple gestation
- 3 months before last menstrual period to day before delivery: healthcare utilization variables, shared environmental factors, maternal comorbidities, maternal medication exposure

Standardized difference (Std.Diff) estimated as $\frac{\bar{X}_{exp}-\bar{X}_{ref}}{\sqrt{\frac{S^2_{exp}+S^2_{ref}}{2}}}$ where $\bar{X}$ represents the sample mean and s^2 the sample variance of the covariate in the exposed (exp) and the reference group (ref).

[a] Variables adjusted for in model 1 but not shown here: maternal age in categories <=19, 20-24, 25-29, 30-34, 35-39, >=40 years (instead, mean age is shown), individual calendar years of delivery (instead, categories are shown), US state of residence (instead, region is shown), adequacy of prenatal care utilization (estimated based on the number of prenatal care visits that occurred accounting for the gestational age at the start of prenatal care and gestational age at birth; adapted from Kotelchuck[1]), timing of prenatal care initiation, timing of first prenatal vitamin dispensing.

[b] Race/ethnicity was determined using information submitted to the Centers for Medicare & Medicaid Services by individual states, which was based on information that had been collected and coded from Medicaid applications.

[c] Information not available in MarketScan.

[d] Adapted from Bateman et al.[2]

[e] Including abnormality of labor forces, umbilical cord complications and obstetrical trauma.

[f] Including artificial rupture of membrane, cercical dilatation and medical induction of labor.

[g] Not considered in adjusted analyses since these variables could be potential mediators of the association between NLA and ASD.

[h] Estimated using a previously validated algorithm based on diagnostic codes for preterm birth.[3]

[i] Including psychosis, sleep disorder, chronic fatigue, behavioral disorder, personality disorder, adjustment disorder, self-inflicted injuries, and other psychiatric disorders.

[j] Including communication/learning disorder, speech/language disorder, coordnination disorder, intellectual disorder.

[k] Including rheumatoid arthritis, psoriasis/psoriatic arthritis, multiple sclerosis, systemic lupus erythematosus, inflammatory bowel disease, addison's disease, graves' disease, sjoegren's syndrome, hashimoto's thyroiditis, myasthenia gravis, autoimmune vasculitis, pernicious anemia and celiac disease.

[l] County-level socioeconomic measures were assessed after linkage of maternal zip codes (only available in MAX) with corresponding FIPS county codes using crosswalk files provided by the US Department of Housing and Urban Development[4]; these FIPS codes were then used to merge the MAX data with data provided

© 2021 Straub L et al. *JAMA Network Open.*

by the US Department of Agriculture which include county-level information on various SES-measures.[5] Metropolitan area is defined as counties in metropolitan areas or counties with urban population of 20,000 or more, adjacent to a metropolitan area; urban area is defined as counties with urban population of 20,000 or more, not adjacent to a metropolitan area, or counties with populations of 2,500 to 19,999 residents; rural area is defined as counties with a population of less than 2,500. Categories of unemployment rate, poverty rate and rate of educational attainment reflect year-specific quartiles.

© 2021 Straub L et al. *JAMA Network Open.*

eReferences.

1. Kotelchuck M. An evaluation of the Kessner Adequacy of Prenatal Care Index and a proposed Adequacy of Prenatal Care Utilization Index. *Am J Public Health*. Sep 1994;84(9):1414-20. doi:10.2105/ajph.84.9.1414

2. Bateman BT, Mhyre JM, Hernandez-Diaz S, et al. Development of a comorbidity index for use in obstetric patients. *Obstet Gynecol*. Nov 2013;122(5):957-65. doi:10.1097/AOG.0b013e3182a603bb

3. Margulis AV, Setoguchi S, Mittleman MA, Glynn RJ, Dormuth CR, Hernandez-Diaz S. Algorithms to estimate the beginning of pregnancy in administrative databases. *Pharmacoepidemiol Drug Saf*. Jan 2013;22(1):16-24. doi:10.1002/pds.3284

4. US Department of Housing and Urban Development's Office of Policy Development and Research, HUD-USPS ZIP Crosswalk Files. Accessed 03/18/2020, https://www.huduser.gov/portal/datasets/usps_crosswalk.html#data

5. US Department of Agriculture - Economic Research Service: Download Data for County-Level Data Sets. Accessed 03/18/2020, https://www.ers.usda.gov/data-products/county-level-data-sets/download-data/

© 2021 Straub L et al. *JAMA Network Open.*
